# HRM and its effect on employee, organizational and financial outcomes in health care organizations

**DOI:** 10.1186/1478-4491-12-35

**Published:** 2014-06-17

**Authors:** Brenda Vermeeren, Bram Steijn, Lars Tummers, Marcel Lankhaar, Robbert-Jan Poerstamper, Sandra van Beek

**Affiliations:** 1 Erasmus University Rotterdam, PO Box 1738, 3000 DR Rotterdam, Netherlands; 2 PwC, PO Box 8800, 3009 AV Rotterdam, Netherlands; 3 Actiz, PO Box 8258, 3505 RG Utrecht, Netherlands

**Keywords:** HRM, Health care, Job satisfaction, Financial outcome, Organizational outcome, Employee outcome, Net margin, Client satisfaction, Sick absenteeism

## Abstract

**Background:**

One of the main goals of Human Resource Management (HRM) is to increase the performance of organizations. However, few studies have explicitly addressed the multidimensional character of performance and linked HR practices to various outcome dimensions. This study therefore adds to the literature by relating HR practices to three outcome dimensions: financial, organizational and employee (HR) outcomes. Furthermore, we will analyze *how* HR practices influence these outcome dimensions, focusing on the mediating role of job satisfaction.

**Methods:**

This study uses a unique dataset, based on the ‘ActiZ Benchmark in Healthcare’, a benchmark study conducted in Dutch home care, nursing care and care homes. Data from autumn 2010 to autumn 2011 were analyzed. In total, 162 organizations participated during this period (approximately 35% of all Dutch care organizations). Employee data were collected using a questionnaire (61,061 individuals, response rate 42%). Clients were surveyed using the Client Quality Index for long-term care, via stratified sampling. Financial outcomes were collected using annual reports. SEM analyses were conducted to test the hypotheses.

**Results:**

It was found that HR practices are - directly or indirectly - linked to all three outcomes. The use of HR practices is related to improved financial outcomes (measure: net margin), organizational outcomes (measure: client satisfaction) and HR outcomes (measure: sickness absence). The impact of HR practices on HR outcomes and organizational outcomes proved substantially larger than their impact on financial outcomes. Furthermore, with respect to HR and organizational outcomes, the hypotheses concerning the full mediating effect of job satisfaction are confirmed. This is in line with the view that employee attitudes are an important element in the ‘black box’ between HRM and performance.

**Conclusion:**

The results underscore the importance of HRM in the health care sector, especially for HR and organizational outcomes. Further analyses of HRM in the health care sector will prove to be a productive endeavor for both scholars and HR managers.

## Background

One of the main goals of Human Resource Management (HRM) is to increase the performance of organizations [1]. Pfeffer [2] emphasized the importance of gaining competitive advantage through employees and noted the importance of several Human Resource (HR) practices necessary to obtain this advantage. Huselid [3] stressed the use of an integrated and coherent ‘bundle’ of mutually reinforcing HR practices over separate ones. Notwithstanding the substantial volume of research on the link between HRM and performance, the exact nature of this relationship within the health care sector remains unclear [4]. This can be considered problematic, as studying HRM in the health care sector and its effect on performance has both practical and academic relevance [5].

However, performance is not a concept that can be easily defined and conceptualized. According to Guest [6] it is better to use the concept of ‘outcomes’ instead of performance. One can then distinguish three different outcomes: 1) financial outcomes (profits, net margin, market share), 2) organizational outcomes (productivity, quality, efficiency, client satisfaction) and 3) HR outcomes (employees’ attitudes and behaviour) [7]. Dyer and Reeves [7] noted that HR and organizational outcomes are more proximal outcomes, for example, closely linked to the HR practices adopted by an organization, whereas financial outcomes are more distant, as they are less likely to be directly affected by HR practices. Moreover, specific HR outcomes are often used as intermediate outcomes that bridge the ‘black box’ between HR practices and financial or organizational outcomes [8].

This multidimensional perspective of outcomes seems especially relevant for health care organizations, as financial outcomes are certainly not the only - or even primary - objective [9]. Notwithstanding the large amount of research on HRM in health care, few studies have explicitly addressed the multidimensional character of performance and linked HR practices to various outcome dimensions [4]. In this article, we therefore add to the literature by examining several outcome dimensions of health care organizations. The research question we will address is as follows: ‘*To what extent are HR practices in health care organizations related to multiple outcome dimensions?’*

First, we will provide a brief background on the link between HRM and outcomes with a specific focus on the health care sector. Next, we will develop several hypotheses. Thereafter, the methods and results of the data analysis are provided. The article ends with a conclusion regarding the effects of HRM on various outcomes in the health care sector.

### HRM and outcomes

Studying the relationship between HRM and performance outcomes is an important research theme [1,10,11]. In an overview article, Boselie *et al*. [12] identified the main research issues within the field. These primarily concern the conceptualization and measurement of the central concepts and several theoretical issues about their relationship. These issues remain important in the contemporary debate [1]. The concept of performance has been discussed above. HRM is commonly defined as a set of employee management activities, but there is no consensus regarding which HR practices should be included in a ‘comprehensive HRM checklist’ [12]. Even more important is the question as to whether one should examine discrete HR practices or employ a systematic HRM approach. According to the systems approach, one should regard interrelated HR practices that affect performance as a ‘synergistic whole’. In this study we follow the systems approach, as this was proven valuable in earlier studies [13].

In addition to conceptualization, there are also important measurement issues concerning HRM. Does one measure HR policies at the company level (for instance by asking HR managers) or at the individual level (practices as experienced by employees)? Nishii and Wright [14] refined this issue by distinguishing among intended, actual and perceived HRM. The notion behind this is that there may be differences within organizations among the HR policy designed by the HR department (intended HRM), the HR practices implemented by line managers (actual HRM) and the perceptions of employees (perceived HRM). This study focuses on perceived HRM, following the Thomas Theorem: if men define situations as real, they are real in their consequences [15]. Thus, if employees believe that specific HR practices are employed in the organization, they will act according to that belief.

An important theoretical issue that has dominated the field in the last decade concerns the precise nature of the mechanism linking HRM and performance outcomes. This issue is called the ‘black box’, i.e., the mediating link between HRM and performance. In recent years, many suggestions have been made regarding the nature of this ‘black box’ [14,16], but most scholars emphasize the perceptions and experiences of employees as the main linking mechanism [12]. HR practices forge a psychological contract between employer and employee that in turn affects these perceptions and experiences. In this article, job satisfaction is used as a mediating variable linking HRM to various outcomes [17,18].

### HRM and outcomes in the health care sector

In the last two decades, several studies on HRM and performance have been conducted in the health care sector [19,20]. In their review of health care studies, Harris *et al*. [4] concluded that HR practices are often related to patient oriented performance outcomes. They also noted the importance of conducting additional research on the ‘black box’ issue. Furthermore, many health care studies relate HRM to organizational and HR related outcomes [21-25]. However, studies focusing on financial outcomes - which have been extensively addressed in the private sector HRM literature - seem rather scarce.

This study focuses on the Dutch care sector (home care, nursing care and care homes). Its contribution concerns two elements discussed in the literature. First, we apply a multidimensional performance perspective, and we will therefore consider three outcome dimensions: financial, organizational and HR. This is innovative because although many health care studies have analyzed care - an organizational outcome - and HR outcomes, financial indicators have received much less attention. Moreover, we are unaware of health care sector studies that have examined the relationship between HRM and these three outcome dimensions simultaneously. The second contribution concerns the ‘black box’ issue. Many studies use employee attitudes as an outcome variable. However, an important interpretation of the ‘black box’ implies that employee attitudes will mediate the link between HRM and performance [13]. Using job satisfaction as indicator of employee attitudes, we will test whether this holds for all three outcome measures considered in this article. This leads to the following three hypotheses:

H1: job satisfaction mediates the relationship between HR practices and financial outcomes in health care organizations.

H2: job satisfaction mediates the relationship between HR practices and organizational outcomes health care organizations.

H3: job satisfaction mediates the relationship between HR practices and HR outcomes in health care organizations.

## Methods

### Data

Before discussing our data, it is important to shortly describe the structure of the Dutch health care sector. In general, the Dutch health care system can be described as a mix of public and private provider agents, mainly based on public funding [26]. More specifically, Dutch health care is divided into short-term care (‘cure’-sector, for instance provided in hospitals) and long-term care (‘care’-sector, for instance provided in nursing homes). This research focuses on organizations that provide long-term care. This includes organizations providing home care, somatic care and psychogeriatric^a^ care and is mainly financed using public funds. Next to this, citizens also pay a relatively small private fee.

A central explanation for the limited number of studies focusing on objective and multidimensional outcome data is that such data are difficult to collect. This study has the advantage of being able to use data from the ‘ActiZ Benchmark in health care’. This benchmark was developed by ActiZ - an important Dutch employer association - in cooperation with PwC - for the period 2010 to 2015. The benchmark measures and compares the performance of three different health care sectors (home care, nursing care and care homes) and contains employee data, client data and financial performance data. We analyzed the data gathered from autumn 2010 to autumn 2011. In total, 162 organizations participated during this period. This is approximately 35% of all organizations providing home care, nursing care and care homes in the Netherlands (http://www.zorggegevens.nl).

The data will be analyzed at the organizational level. Thus, data collected at the employee or client level will be aggregated. Other variables, such as financial performance indicators, do not need to be aggregated, as they are (only) available at the organizational level. With respect to financial outcomes, we will consider the net margin. With respect to organizational outcomes, we will focus on client satisfaction, and absence due to sickness will be considered to capture HR outcomes. Job satisfaction - which also can be regarded as an (proximal) HR outcome - will be used as a ‘black box’ variable mediating the relationship between HR practices and outcomes. The measurement of HR practices is discussed below.

First, most financial performance data on health care organizations are publicly available and based on annual reports. This information is stored in databases (available at http://www.zorggegevens.nl and http://www.jaarverslagenzorg.nl) (in English: healthcare information and annual reports). We discussed this information with an accountant from PwC. To gather employee data, a questionnaire was distributed to all employees, and a total^1^ of 61,061 individuals completed the survey, resulting in a response rate of 42%. Only the responses of employees with direct interactions with clients were used in our analysis (job functions such as nursing, care, client-related domestic support and occupational therapy), due to their relationship with the organizational outcome (client satisfaction). This resulted in a database of 48,145 employees. Within this employee database, each question was answered by at least 90.7% of the respondents. Of the valid respondents, 92% were women. This is consistent with Dutch averages for employees in home care, nursing care and care homes, which is predominantly a female profession [27]. As age is subdivided into categories in our study, we could only say something about the predominant age category. The predominant age category is 46 to 55 years (36.9%) which suggests that the average age is slightly above the average age of 41 years [27]. Clients were surveyed using the Client Quality Index (CQi) for long-term care [28,29]. The CQi employs a stratified sampling method, through which an independent agency surveys a representative client sample for each organization. Three groups are constructed: home care clients, somatic care clients (in nursing homes or care homes) and psychogeriatric care clients (in nursing homes or care homes). Home care clients are asked to complete a survey; somatic clients are interviewed using a survey as a guide. For psychogeriatric clients (suffering from cognitive issues such as dementia), an authorized representative completes a survey.

To ensure the comparability of the employee data with the client and financial performance data, we only included organizations with information in all three databases. This resulted in a database with 85 organizations.

### Measurement

The dataset constructed as described above has the potential to increase our understanding of the relationship among HR practices, job satisfaction and outcomes. However, it also has limitations. The data are not gathered with academic objectives in mind; instead, its primary goal is to be practically useful for the organizations involved. This implies that items used in this study are only partly based on validated scales and existing theory. To determine the reliability of the scales, we have computed reliability statistics where possible. Cronbach’s Alpha is used as a measure of reliability. It indicates how consistently the observed variable measures the latent dimension (prescribed norm is > .70).

#### HR practices

The employee questionnaire contains five indicators that are often used in HRM and performance research: training and development, performance related pay, teamwork, job design, and autonomy. In the overview article by Harris *et al*. [4] the measurement of HRM in health care is discussed. They stated that HR practices that should be adopted in HRM systems incorporate high performance work practices found to have had a positive effect on performance in other sectors (the so-called best practices) without derogating the specific health care context. The first two indicators included by us are the most frequently used in research [12]. The other three also score relatively high on the list of the most common practices (ranked 5, 10 and 11) [12]. However, HRM and performance research exhibits little consistency in the selection of HR practices to measure HRM. Boselie *et al*. [12] analyzed 104 important HRM and performance studies and identified as many as 26 different HR practices used in different studies. No single agreed, or fixed, list of HR practices or systems of practices exists to measure HRM [30,31]. Nevertheless, a certain consensus regarding the measurement of HRM has emerged in the academic literature on HRM and performance during the last decade. More than half of the articles published after 2000 made use of AMO (Ability, Motivation and Opportunity) theory [30]. AMO theory proposes that an HRM system should be designed to meet employees’ needs for skills and motivation and, after meeting those needs, provide them with opportunities to use their abilities in various roles [32]. The underlying idea is that employees will perform well if they have the requisite abilities, when they are motivated and when they obtain the opportunity to profile themselves [32]. By using the five HR practices indicated above, all three dimensions of AMO theory are covered. Lepak *et al*. [33] have listed concrete HR practices that influence employees’ AMO. In this respect, training and development are expected to improve employees’ abilities (A), performance related pay is an HR practices to motivate employees to perform (M), and teamwork, job design and autonomy are HR practices that are considered as opportunities to perform [30]. These five HR practices are also regularly part of the measurement of HRM in health care studies [21,24,34].

*Training and development* was measured using three items. A sample item is: ‘My organization pays enough attention to my career’. Responses were given using a five-point Likert scale (‘totally disagree’ to ‘totally agree’). All standardized loadings were greater than .5. Cronbach’s alpha was .77.

*Performance related pay* was measured using one item: ‘My organization provides additional financial rewards to employees with exceptional performance’. Responses were provided on a four-point Likert scale ranging from ‘never’ to ‘always’.

*Teamwork* was measured using two items. A sample item is: ‘Our organization encourages me to work together with other work units/teams or individuals within the organization’; (four-point Likert scale, ‘never’ to ‘always’). All standardized loadings were greater than 0.5, and they were all statistically significant. Correlation between the two items is .547 (*P* < .001).

*Job design* was measured using three items. A sample item is: ‘My tasks are clear’ (four-point Likert, ‘never’ to ‘always’). All standardized loadings were greater than 0.5, and they were all statistically significant. Cronbach’s alpha was .85.

Four items were used to measure *autonomy*. A sample item is: ‘I can make decisions independently’ (four-point Likert, never to always). All standardized loadings were greater than .5, and they were all statistically significant. Cronbach’s alpha was .76.

As stated above, we followed the systems approach and therefore combined the five indicators into one HR system variable. As our analysis is at the organizational level, we aggregated the employee data. In this type of analysis, only variables with sufficient variance across organizations are included. To determine whether the data could be aggregated, the intraclass correlation (ICC) was computed. Aggregation is permissible when the variance between groups is larger than the variance within groups. For all HR practices, aggregation was permissible: training and development (F = 11.400, *P* < 0.01), performance related pay (F = 20.455, *P* < 0.01), job design (F = 7.728, *P* < 0.01), teamwork (F = 14.240, *P* < 0.01), autonomy (F = 8.391, *P* < 0.01), as was the overall HRM variable (F = 9.667, *P* < 0.01).

#### Job satisfaction

Job satisfaction was measured by one item: ‘I enjoy going to work’ (F = 6.586, *P* < 0.01) (five-point Likert, ‘never’ to ‘always’). Nagy [35] noted that measuring job satisfaction with a single item ‘is more efficient, is more cost-effective, contains more face validity, and is better able to measure changes in job satisfaction’.

#### Financial outcome

The net margin is defined as the ratio of a firm’s net profits to its total revenues. It indicates what share of each euro/dollar earned is translated into profit. It is stated as a percentage:

Netprofit/Totalrevenues*100=Netmargin

#### Organizational outcome

The organizational outcome is measured by focusing on client satisfaction. Clients were asked about their satisfaction with the treatment they received. This indicator consists of five items. A sample item is: ‘Do the caregivers have enough time for you?’ (four-point Likert, ‘never’ to ‘always’). We must note that the Association of Client Quality only provides aggregated scales, partly because of privacy issues. Thus, the reliability statistics and ICC cannot be computed. However, the robustness of the CQi - which is most often analyzed at the organizational level - shows that aggregation seems appropriate [25,26].

#### HR outcome

The HR outcome measure considered is absence due to sickness. Absence due to sickness can be considered a key HR outcome as the decision of employees to be absent affects the available human resources and is a critical success factor for the continuation of work processes within the organization (for example, see [36]). Absenteeism due to sickness is calculated in percentages, using a standard formula developed by Vernet [37]. In brief: for every employee, each day he/she calls in sick is multiplied by the part-time factor and disability factor pertaining to that day. These days are then summed and divided by the total number of working days. Maternity leave is excluded. This is calculated for the organization as a whole.

#### Control variables

We also included control variables, such as gender (1 = female) and age (1 = up to 25 years; 2 = 26 to 35 years; 3 = 36 to 45 years; 4 = 46 to 55 years; 5 = 56 years and older). Furthermore, we included diversity of care to determine whether the relationship among the variables differs for organizations employing a diverse set of care activities as supposed to more specialized organizations. It ranges from a minimum of one to a maximum of six as there are six different forms of care in our sample: hospital care, extramural residential care, extramural personal care, day activities, maternity care and youth care^b^.

### Method of analysis

The hypotheses were tested using structural equation modeling (SEM) with Robust Maximum Likelihood estimation. SEM allows us to test the full conceptual model simultaneously. Furthermore, SEM allows us to simultaneously analyze the direct and indirect relationships among the independent and the dependent variables. Finally, SEM also enables us to compare different models [38]. We used AMOS version 21 IBM SPSS (see http://www-03.ibm.com/software/products/nl/spss-amos) to develop the SEM model.

As our hypotheses include mediation effects, we employed bootstrapping [39]. This method estimates the parameters of a model and their standard errors strictly from the sample without reference to any theoretical sampling distribution. In our study, we created 200 samples (with replacement) from the available observed sample.

## Results and discussion

Table 1 presents the means, standard deviations and correlations of the variables. As perception variables are measured on various scales (1 to 5 or 1 to 4), we recoded them into a 1 to 10 scale to ease interpretation. The results show that employees perceive a relatively large number of HR practices (M = 6.08 on a 1 to 10 scale). Employees are on average satisfied with their jobs (M = 8.15). Client satisfaction is also quite high: 8.63. With respect to absence due to sickness, the average score is .06 (6%). Finally, the average value for the net margin was .03, showing that for each 100 euros of revenue, 3 euros accrue as profits. Furthermore, the correlations show that HR practices are related to the outcomes as expected. For instance, HR practices are positively and significantly related to client satisfaction. As some of the bivariate correlations are in the medium to high range, we conducted multicollinearity tests. The variance inflation factor (VIF) values were all well within the acceptable range, with the highest being 2.05 [40]. Thus, our results are not adversely affected by multicollinearity.

**Table 1:** Means, standard deviations, and correlations (N = 85)

	**Mean**	**SD**	**1**	**2**	**3**	**4**	**5**	**6**	**7**
1. HRM	6.08	.433							
2. Job satisfaction	8.15	.422	.725^b^						
3. Sick absenteeism	.06	.017	-.494^b^	-.424^b^					
4. Client satisfaction	8.63	.346	.273^a^	.286^a^	-.381^b^				
5. Net margin	.03	.035	.267^a^	.188	-.177	.187			
6. Diversity of care	4.34	.699	-.333^b^	-.278^b^	.345^b^	-.246^a^	-.153		
7. Age	3.31	.293	-.508^b^	-.427^b^	.457^b^	-.133	-.057	.229	
8. Gender	.92	NA	.155	.137	-.276^a^	.379^b^	.110	-.071	-.065

^a^*P* < .05; ^b^*P* < .01.

**Figure 1:**
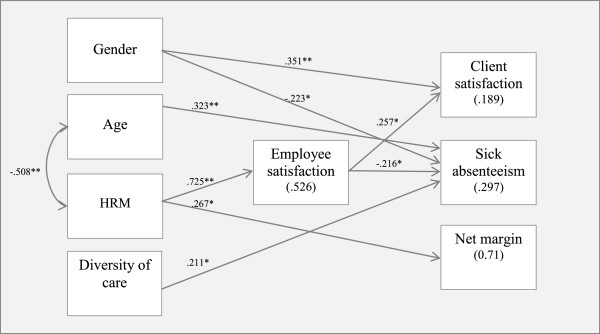
Result of Structural Equation Modeling.

To test the proposed relationships, a structural equation model was developed, as shown in Figure 1. Only the statistically significant relationships are described (*P* < .05). The numerical scores on all lines indicate standardized regression coefficients (beta), and the scores in brackets are the explained variance. The overall model fit was tested using several indices. The model fit values were CMIN 24.146 (df 19, *p* .191) and .962 (comparison fit index (CFI)), implying that the model had a very good fit. Additionally, the root mean square error of approximation (RMSEA), with a value of .057, also indicated that the model had a good fit.

We can now discuss the hypotheses in detail. First, we tested the hypothesis that job satisfaction mediates the relationship between HR practices and financial outcomes in the Dutch care sector. The results show that this indirect effect is not statistically significant (see Table 2). We therefore reject the first hypothesis concerning a mediating effect. This could imply that the effect of HR practices on financial performance is direct and not mediated by job satisfaction. The results indeed show a positive and significant relationship between these variables (β = .267, *P* < .05), implying that a greater use of HR practices is directly related to improved financial outcomes.

**Table 2:** Indirect effects of Human Resource Management (HRM) on outcomes mediated by job satisfaction

	**HRM**
H1: Financial outcome (net margin)	-.008
H2: Organizational outcome (client satisfaction)	.186^b^
H3: HR outcome (sick absenteeism)	-.157^a^

^a^*P* < .05; ^b^*P* < .01.

The second hypothesis proposed that job satisfaction mediates the relationship between HR practices and organizational outcomes. The results show that this is indeed the case. Therefore, our second hypothesis is supported by the data.

Finally, we tested the hypothesis that job satisfaction mediates the relationship between HR practices and HR outcomes in the Dutch care sector. The results indeed show that the indirect relationship between HRM on the HRM outcome sick absenteeism is significant. Therefore, our third hypothesis is also supported by the data.

The final step in the analysis was the examination of the control variables. In organizations with more female employees, clients are more satisfied with the delivery of services. Moreover, the percentage absence due to sickness is lower in these organizations. With respect to age, the results show that absence due to sickness is higher in organizations in which the average age is relatively high. Finally, the diversity of care is positively associated with absence due to sickness. In other words, organizations engaging in a diverse set of care activities have more absence due to sickness than more specialized organizations.

Finally, model validity was achieved through cross-model validation. Camilleri [41] suggests pursuing cross-validation in three phases. In the first phase, the data are divided into two data sets. One dataset consists of a random selection of 20% of the data collected from respondents; the second dataset consists of a random selection of 80% of the data collected. In the second phase, SEM via path analysis that calculates the structural fit index (measured by R^2^) is conducted for both datasets. The third phase consists of examining the differences between the calculated structural fit indices obtained for each dataset. The extent of model validity is determined by the similarity in the variance accounted for by each dataset. The results of the cross-model validation are presented in Table 3. As the differences in the explained variance are small, the cross-model validation provided satisfactory results.

**Table 3:** **Results of cross-model validation showing R**^**2 **^**for the three samples**

**Predicted variable**	**Full Sample**	**20% Sample**	**80% Sample**	**Difference in R**^**2**^**between the 20 and 80% Samples**
Job satisfaction	.526	.460	.556	-.096
Financial outcome	.071	.020	.044	-.024
Organizational outcome	.189	.145	.212	-.067
HR outcome	.297	.294	.267.	.027

## Conclusion

The main contributions of this study to the literature on HRM and performance in the health care sector concerns the use of a multidimensional performance perspective. In this respect, we examined three different outcomes: financial (net margin), organizational (client satisfaction), and HR (sickness absence). The analysis includes job satisfaction, which can be regarded as a ‘black box’ variable: a mediating variable connecting HR practices and performance.

The results confirm the basic notion that HRM and performance within the health care sector are linked. Our final SEM model shows that HRM is - directly or indirectly - linked to all three outcomes. When organizations apply - according to their employees - more HR practices, this is associated with greater client satisfaction, less sickness absence, and a better net margin. With respect to organizational and HR outcomes, the hypotheses regarding the mediating effect of job satisfaction are confirmed. This is in accordance with the perspective that employee attitudes are an important component of the ‘black box’ between HRM and performance. In this respect, our study showed that higher job satisfaction is associated with higher organizational performance. More specifically, in line with the assumption, our research showed a positive association between employee satisfaction and customer satisfaction because if employees are satisfied with their jobs, they are likely to behave toward customers in ways that yield positive service experiences. A more extensive use of HR practices leads to more satisfied employees. This greater satisfaction ‘reflects’ on the clients, as satisfied employees will do more for them [42]. Moreover, satisfied workers are less likely to call in sick than less satisfied workers.

HR practices are directly related to financial outcomes, although the explained variance is small. Furthermore, we found that job satisfaction does not mediate the relationship between HRM and net margin. As we mentioned in the introduction, financial outcomes are a distant outcome of HRM. In fact, the literature about strategic management informs us that organizations can use different strategies to achieve their objectives [43]. In addition to a high performance strategy, organizations can also employ a low cost strategy [44]. Boxall and Purcell [45] describe the ‘mass service market’ - which includes care - as a ‘service market with some quality differentiation’. Organizations can follow various strategies to become (financially) successful. One possible strategy implies investing in employees, which will likely result in more satisfied employees. Another strategy implies cutting costs, which will result in reduced investments in employees and (most likely) less satisfied employees. The finding that HRM has a direct effect on financial outcomes may be because a low cost strategy also implies the use of certain HR practices, for instance performance management. It can thus lead to financial success without positively affecting the satisfaction of employees.

We conclude this article by presenting some limitations. An important limitation of this research - but also of many other studies in this area - is the hidden assumption that the same mix of HR practices will work for all organizations. Therefore, the inclusion of HR strategy in research designs will be an important addendum.

The possibility of considering various data sources (employee, client and ‘objective’ performance data) is an important - and unique - advantage of this study. However, it also has some drawbacks. The scales used are not based on previous academic literature. In further research, validated scales should therefore be employed. Moreover, a disadvantage of using secondary data is that not all the desired research concepts were covered in the data.

A further limitation is the sample size. Although the underlying dataset is large, the data were aggregated at the level of 85 health care organizations. This could be considered quite low. However, Bentler and Chou [46] recommended a ratio of sample size to free parameters of at least 5:1. In our analysis, the model tested was simple, and the ratio of the number of free parameters to the number of cases did not fall below under 5:1. Related to this, several studies using SEM with a small sample size are available [47-49]. Nevertheless, future studies might attempt to replicate the findings using larger sample sizes.

Furthermore, the results of this study should be interpreted in light of the study’s context and sample. The study was conducted in the Netherlands, which features a social health insurance scheme in health care financing and a mix of public and private provider organizations in health care provision [26]. This is in line with other ‘Bismarck’ countries, such as Belgium, Germany and France [26]. It would be interesting to replicate our study to test the proposed model in other countries using different kinds of health care systems.

In conclusion, our empirical results underscore the importance of HRM in the health care sector. We can state that HRM makes a difference, especially for HR and organizational outcomes. Its impact on financial performance is less strong. Job satisfaction links HR practices and organizational and employee outcomes. In conclusion, further analyzing HRM in the health care sector will be a productive endeavour for both researchers and practitioners.

## Endnotes

^a^In some countries this terminology is no longer used. However, according to the organization of the care in the Netherlands, ‘psychogeriatric care’ is supposed to be the correct terminology.

^b^In this variable, more distinct forms of care are included than are analyzed in our study. We use this variable, however, as a proxy for the complexity of the organization.

## Abbreviations

AMO: Ability, Motivation and Opportunity; CFI: comparison fit index; CQi: Client Quality Index; HR: Human Resources; HRM: Human Resource Management; ICC: intraclass correlation; RMSEA: root mean square error of approximation; SEM: structural equation modeling; VIF: variance inflation factor.

## Competing interests

The authors declare that they have no competing interests.

## Authors’ contributions

BS, BV and LT have contributed to the manuscript by drafting the manuscript (all sections). BV has furthermore contributed by conducting the statistical analyses. RJP and ML have contributed by designing the datasets which form the foundation of the manuscript and by acquisition of data. SvB has contributed by drafting the manuscript and revising it critically. All authors approved the final manuscript.
